# Increased gastrointestinal blood flow: An essential circulatory modification for euryhaline rainbow trout (*Oncorhynchus mykiss*) migrating to sea

**DOI:** 10.1038/srep10430

**Published:** 2015-05-22

**Authors:** Jeroen Brijs, Michael Axelsson, Albin Gräns, Nicolas Pichaud, Catharina Olsson, Erik Sandblom

**Affiliations:** 1 Department of Biological and Environmental Sciences, University of Gothenburg, Gothenburg, Sweden; 2 Department of Animal Environment and Health, Swedish University of Agricultural Sciences, Skara, Sweden

## Abstract

The large-scale migrations of anadromous fish species from freshwater to seawater have long been considered particularly enigmatic, as this life history necessitates potentially energetically costly changes in behaviour and physiology. A significant knowledge gap concerns the integral role of cardiovascular responses, which directly link many of the well-documented adaptations (*i.e.* through oxygen delivery, water and ion transport) allowing fish to maintain osmotic homeostasis in the sea. Using long-term recordings of cardiorespiratory variables and a novel method for examining drinking dynamics, we show that euryhaline rainbow trout (*Oncorhynchus mykiss*) initiate drinking long before the surrounding environment reaches full seawater salinity (30–33 ppt), suggesting the presence of an external osmo-sensing mechanism. Onset of drinking was followed by a delayed, yet substantial increase in gastrointestinal blood flow through increased pulse volume exclusively, as heart rate remained unchanged. While seawater entry did not affect whole animal energy expenditure, enhanced gastrointestinal perfusion represents a mechanism crucial for ion and water absorption, as well as possibly increasing local gastrointestinal oxygen supply. Collectively, these modifications are essential for anadromous fish to maintain homeostasis at sea, whilst conserving cardiac and metabolic scope for activities directly contributing to fitness and reproductive success.

Anadromy, a distinctive life-history trait involving the migration across the freshwater-ocean boundary, provides considerable adaptive and selective advantages for many fishes^1^,^2^,^3^. Higher productivity in oceans compared to freshwater habitats at temperate latitudes may provide richer food sources and enhanced food intake for migrating anadromous fish^1^. Increased food intake and the consequent contribution of growth to life history traits have been shown to be selectively advantageous for the lifetime reproductive success of an individual^2^. However, a range of potentially energetically costly physiological and behavioural changes must occur for fish to survive contrasting osmotic environments; all of which have shaped the evolution and ecology of anadromous fishes^2^,^3^,^4^.

While most fishes only tolerate relatively small changes in environmental salinity, euryhaline teleosts such as rainbow trout (*Oncorhynchus mykiss*) can tolerate and acclimate to a broad range of salinities. In freshwater, a dilute ionic and osmotic medium, fish must hyper-osmoregulate to counter the continual loss of salts and entry of water across their permeable body surfaces^5^. Mechanisms of hyper-osmoregulation include active branchial and gastrointestinal ion uptake^5^, high glomerular filtration rates and urine flows, while minimizing drinking rate and renal salt loss^6^. Conversely, in marine environments, fish hypo-osmoregulate to counter the osmotic loss of water and diffusional gain of salts^7^. Generally, exposure to increasing salinity requires pre-existing mechanisms to respond to the changing osmotic conditions. During the first 1-2 days of acute exposure, often referred to as the ‘crisis period’^8^, drinking rates typically increase 10 to 50-fold^7^,^9^. Gastrointestinal absorption of Na^+^ and Cl^-^ from imbibed seawater drives water uptake^10^, and coincides with increased branchial and renal excretion of monovalent and divalent ions, respectively^8^,^11^. A longer regulatory or stabilization phase follows, which consists of permanent osmoregulatory changes such as increased branchial and intestinal Na^+^/K^+^-ATPase expression and activity^6^,^7^.

The physiological and behavioural changes of fishes transitioning from freshwater to seawater have been suggested to increase energy expenditure^4^. However, literature on changes in oxygen consumption (*i.e.* standard metabolic rate, SMR) at different salinities is conflicting with respect to the energetic costs of osmotic and ionic regulation. Estimates of the energetic cost for osmoregulation range from virtually none to approximately one third of the SMR^12^. In addition, the correlation between acclimation or acute exposure to varying environmental salinities and SMR remains highly variable^12^.

Metabolic and osmoregulatory changes associated with the transition from freshwater to seawater could be expected to coincide with circulatory adjustments. Increased environmental salinity may also indirectly affect cardiovascular function through dehydration, resulting in decreased blood volume and increased viscosity due to haemoconcentration^13^,^14^. Knowledge concerning the cardiovascular responses of fish exposed to changes in environmental salinity remains scarce. One study estimated that cardiac output in freshwater acclimated rainbow trout increases by approximately 30% when acutely exposed to seawater^15^. However, this study only examined circulatory responses for 24 h following seawater transfer and it remains unknown how prolonged exposure may affect the response. Moreover, seawater entering the gut during drinking likely requires increased gastrointestinal processing (*e.g.* water and ion absorption). This would potentially entail an increased gastrointestinal blood flow (GBF) in order to transport absorbed ions and increase oxygen supply as metabolic demands increase in the active gastrointestinal tissues^16^,^17^. Even so, despite the previously suggested benefits of gastrointestinal hyperemia during seawater transfer^16^,^18^, gut blood flow responses in fish exposed to different salinities remain completely unexplored.

The overall aim of the present study was to examine the time course of cardiorespiratory changes in rainbow trout during an acute transition from freshwater to seawater. Specifically, we hypothesized that upon a transition to seawater, rainbow trout would exhibit an increase in GBF and SMR to transport absorbed ions and water, as well as supply oxygen and nutrients to a more metabolically active gastrointestinal tract. We therefore recorded changes in whole animal oxygen consumption and gastrointestinal blood flow. Additionally, the dynamics of the drinking response was determined using a novel method measuring the conductivity of fluids inside the stomach. The knowledge gained from this study is essential for understanding physiological and energetic processes operating in fish migrating from freshwater to seawater, and ultimately the environmental selection pressures and physiological constraints that have shaped the evolution and ecology of anadromy in fishes.

## Results

### Gastrointestinal blood flow increases during seawater exposure

**Figure 1:**
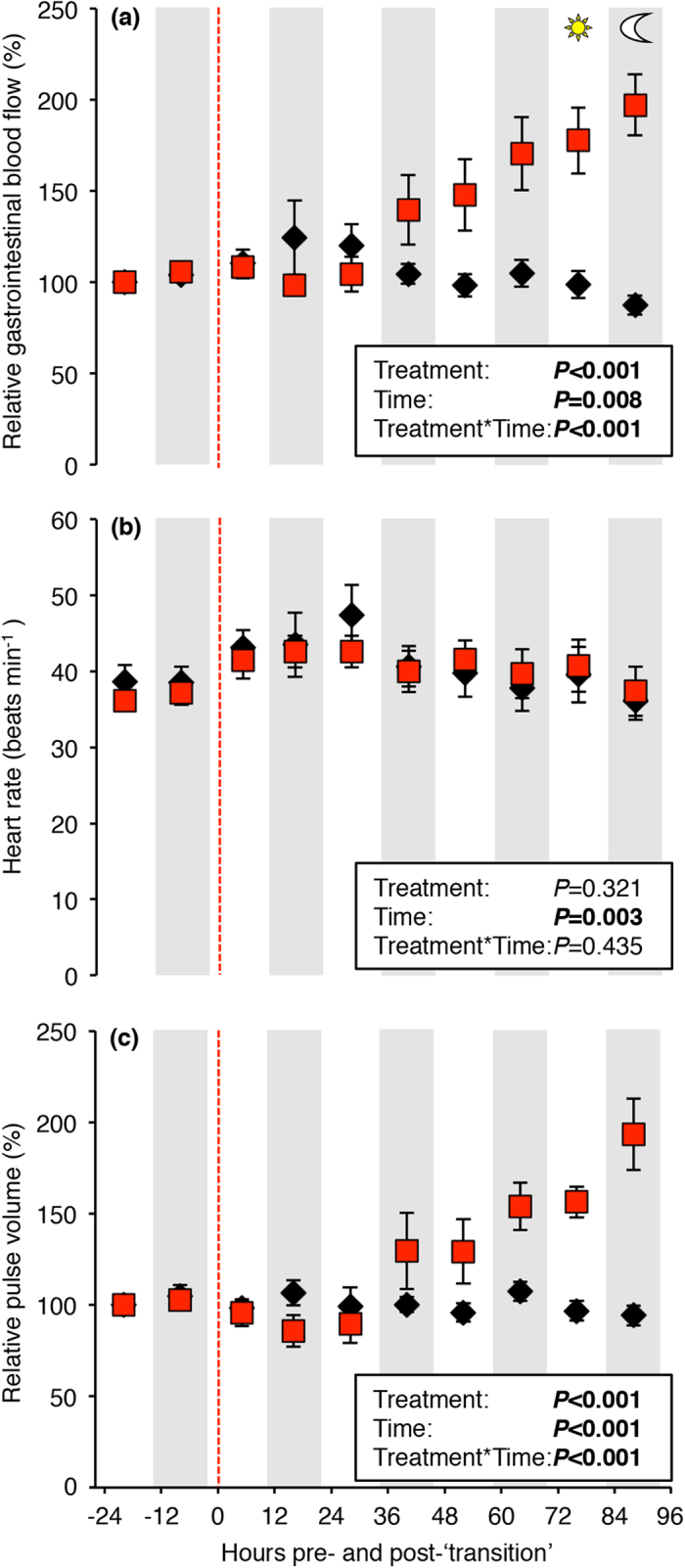
Cardiovascular variables of instrumented rainbow trout transitioning to seawater or remaining in freshwater. **** Relative gastrointestinal blood flow (**a**), heart rate (**b**) and relative pulse volume (**c**) of rainbow trout transitioning to seawater (30–33 ppt, red squares) or remaining in freshwater (<0.1 ppt, black diamonds) at 13.5 ± 0.1 °C. The transition to seawater resulted in a significant increase of gastrointestinal blood flow and pulse volume over the experimental period, whereas, heart rate was not significantly affected by a transition to seawater. Red hatched line represents a transition to seawater or a ‘simulated water change’ (*i.e.* fish remained in freshwater) at 0800 h. Statistical analyses generated using a linear mixed model are summarized in figure box. Data are presented as means ± s.e.m. (n = 8).

GBF was significantly affected by a transition to seawater (F = 26.48, *P* < 0.001), time (F = 7.19, *P* = 0.008) and their interaction (F = 50.48, *P* < 0.001, Fig. 1a). Approximately 36 h after the transition to seawater, GBF began to steadily increase and reached maximum relative values of 197 ± 17% after 96 h.

Heart rate fluctuated between approximately 35 and 50 beats min^−1^ throughout the experimental period and was significantly affected by time (F = 8.89 *P* = 0.003), but not by a transition to seawater or their interaction (Fig. 1b).

In contrast, pulse volume was significantly affected by a transition to seawater (F = 24.24, *P* < 0.001), time (F = 30.11, *P* < 0.001) and their interaction (F = 38.33, *P* < 0.001, Fig. 1c). The pulse volume showed a response profile that closely mirrored that of GBF (compare Fig. 1a,c), increasing after 36 h in seawater and reaching a relative pulse volume of 193 ± 17% at 96 h. In freshwater, pulse volume did not significantly change throughout the experimental period (Fig. 1c).

### Osmoregulation in seawater does not incur additional energetic costs

**Figure 2:**
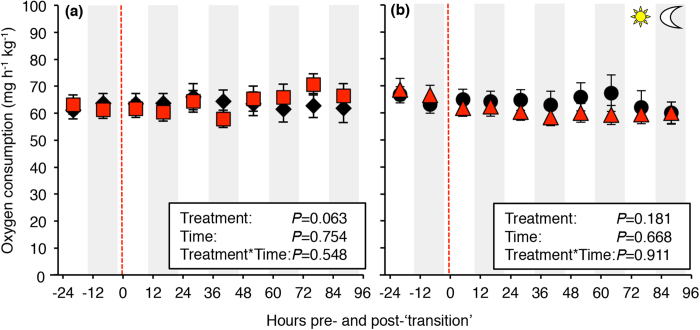
Standard metabolic rate (SMR) of surgically instrumented and uninstrumented rainbow trout transitioning to seawater or remaining in freshwater. **** Standard metabolic rate (SMR) in fish instrumented with a Doppler flow probe around the coeliacomesenteric artery (**a**) and uninstrumented fish (**b**) transitioning to seawater (30–33 ppt, red symbols) or remaining in freshwater (<0.1 ppt, black symbols) at 13.5 ± 0.1 °C. SMR was not significantly affected by a transition to seawater in either instrumented or uninstrumented fish. Red hatched line represents a transition to seawater or a ‘simulated water change’ (*i.e.* fish remained in freshwater) at 0800 h. Statistical analyses generated using a linear mixed model are summarized in figure box. Data are presented as means ± s.e.m. (n = 8).

SMR typically ranged between 55 and 70 mg h^−1^ kg^−1^ throughout the experimental period and was not significantly affected by a transition to seawater, time or their interaction in either instrumented or uninstrumented fish (Fig. 2a,b). Moreover, the surgical implantation of gastrointestinal blood flow probes did not significantly affect SMR (*P* = 0.826 for fish remaining in freshwater, *P* = 0.241 for fish exposed to seawater).

### Rapid up-regulation of drinking rate in seawater

**Figure 3:**
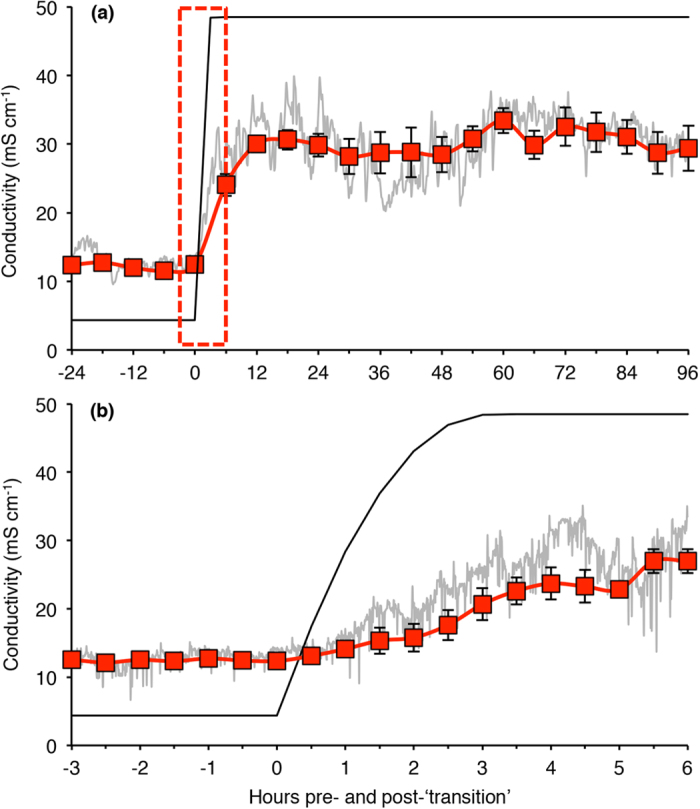
Drinking dynamics in rainbow trout transitioning to seawater. **** Temporal changes in stomach fluid conductivity in rainbow trout (red squares, n = 4) transitioning from freshwater to seawater (**a**). A magnified section displaying changes in stomach fluid conductivity during the transition period (**b**). The black line represents the conductivity of the ambient environment and the grey line is a raw trace from a representative fish. Data are presented as means ± s.e.m. (n = 4).

Prior to the transition to seawater, the conductivity of the stomach fluid in rainbow trout remained stable at 12.2 ± 0.2 mS cm^−1^, which was approximately three-fold higher than the ambient environment (Fig. 3a). Increasing salinity of the surrounding water resulted in a rapid increase of the conductivity of stomach fluid in all individuals, however, the timing of the increase varied considerably between individuals (ranging between 16 and 140 min after the transition was initiated). Nevertheless, stomach fluid conductivity of all individuals slowly, but steadily, increased long before the salinity of the ambient environment reached full strength seawater (Fig. 3b). After approximately 12 h, the conductivity had stabilized around 25 and 35 mS cm^−1^, which was approximately 60% of the conductivity of the ambient seawater environment.

## Discussion

Rapid acclimation to increasing salinity through physiological and behavioural modifications is beneficial for euryhaline fish migrating to the sea, as it may permit the immediate resumption of feeding and growth^2^,^4^. Many of these modifications have been documented, but descriptions of circulatory responses to water salinity changes in fish are conspicuously absent from the literature. Thus, this is the first study examining gastrointestinal blood flow responses in fish acclimating to increased water salinity.

Due to the relatively long post-surgical recovery (72 h) in the present study, the rainbow trout had a low resting heart rate prior to the start of the experiment. In fact, resting heart rates are similar to values reported for trout using non-invasive methods for cardioventilatory recordings indicating very low post-operative stress^19^,^20^. Additionally, the surgical implantation of gastrointestinal flow probes did not significantly affect SMR as both uninstrumented and instrumented fish had similar values ranging between 55 and 70 mg h^−1^ kg^−1^. Collectively, this suggests that the data obtained from instrumented fish in the present study may closely reflect what is occurring in uninstrumented fish.

It is well documented that rainbow trout and other salmonids significantly increase drinking rates upon transition to seawater, with maximum reported values approaching 25 ml kg^−1^ h^−1^ 18,21,22. However, the traditional method used to measure drinking in salmonids (*i.e.* dissolving a marker in the environmental water and measuring subsequent gastrointestinal uptake) does not provide high temporal resolution of the drinking dynamics of an individual fish. By adopting a novel method assessing drinking via conductivity measurements, we show that rainbow trout do not drink whilst in freshwater as conductivity of stomach fluids was stable and approximately three-fold higher than the surrounding water. Even so, a drinking response (*i.e.* an increase in stomach fluid conductivity) was initiated in all individuals long before the salinity of the ambient environment had reached full strength seawater salinity (30–33 ppt). Thus, similar to the Japanese eel (*Anguilla japonica*)^23^, rainbow trout started drinking much faster than what can be accounted for by the dipsogenic effects of decreased blood volume or hypotension caused by dehydration^24^. This suggests that the drinking response was, at least initially, triggered by stimulation of external osmo-receptors that provide afferent input to the central nervous system to initiate a drinking response^25^. Additionally, we could show that conductivity of fluids entering the stomach was always less than ~60% of the surrounding seawater. This provides additional evidence that substantial esophageal desalinization (*i.e.* NaCl absorption) of imbibed seawater, as well as further dilution in the stomach (via minor fluxes of solutes and water), reduces seawater osmolarity and facilitates subsequent intestinal water absorption^7^,^26^,^27^,^28^.

In contrast to the rapid drinking response, GBF displayed a delayed response in seawater. Approximately 36 h after the transition, GBF began to increase and reached maximum values after 96 h, which were two-fold higher than those measured in freshwater. Although previous studies have hypothesised that gastrointestinal perfusion may be important for increasing the hypo-osmoregulatory capacity of euryhaline fish^16^,^18^, this is the first direct recording of GBF during increasing environmental salinity to substantiate this idea. The magnitude of the increase in GBF was similar to the substantial increase that occurs after feeding in rainbow trout^29^. This suggests that gastrointestinal hyperemia plays an integral role in maintaining osmotic homeostasis of fish in seawater. In addition to the obvious benefits of supplying oxygen and nutrients to metabolically active gastrointestinal tissues^17^, the increased GBF in seawater is most likely important for transporting absorbed ions and water from the gastrointestinal tract to their respective sites for excretion or utilization^18^. The delayed response of GBF in rainbow trout may be associated with the time lag that occurs between increased Na^+^/K^+^-ATPase expression and the adaptive increase in branchial and intestinal Na^+^/K^+^-ATPase activity after a transfer to seawater^8^,^30^. Interestingly, studies on a range of salmonid species have shown that increased branchial and intestinal Na^+^/K^+^-ATPase activity occurs 24–72 h after seawater transfer^8^,^30^,^31^,^32^. This approximately coincides with the progressive increase in GBF observed in the present study, indicating that it may be necessary for the gills and the gastrointestinal system to transform from hyper- into hypo-osmoregulatory organs prior to increasing GBF for water uptake and transportation of ions for subsequent excretion. Furthermore, the timing of increased Na^+^/K^+^-ATPase activity and GBF roughly coincides with the beginning of a gradual return of osmotic balance following the initial ‘crisis period’ previously reported in trout^21^,^22^,^30^,^33^. These studies showed that following a transition to seawater, fish displayed increasing concentrations of blood electrolytes and decreasing body water content for ~2 days before plasma osmolality gradually decreased and eventually stabilized at values previously observed in freshwater.

Previous studies on euryhaline teleosts in seawater have shown exacerbated dehydration during exercise^16^,^34^. This is typically interpreted as a result of the ‘osmo-respiratory compromise’, where increased gill blood flow and hypertension during exercise leads to increased water loss to the surrounding hyperosmotic environment^35^. Since GBF typically decreases in swimming fish^36^,^37^,^38^, a possible fascinating implication of the role of GBF in maintaining fluid balance and osmotic homeostasis is that impaired intestinal water absorption may, at least in part, explain the dehydrating effects that fish swimming in seawater experience^16^. This line of argumentation is further supported by circumstantial evidence from chronically exercised trained Chinook salmon (*Oncorhynchus tshawytscha*) that were better able to both preserve their GBF, as well as maintain plasma osmolality, during swimming relative to untrained conspecifics^16^,^37^.

A previous study on rainbow trout acutely exposed to seawater, estimated cardiac output using the Fick equation and suggested a 30% increase in seawater that was primarily determined by increased cardiac stroke volume^15^. GBF can be modified by changes in overall cardiac output, systemic and gastrointestinal vascular resistances that redirect blood flow towards or away from the gastrointestinal tract or combinations of these factors^17^. Thus, the most parsimonious explanation for the observed pulse volume mediated increase in GBF observed in this study is most likely through a combined increase in cardiac stroke volume and reduced gastrointestinal vascular resistance, especially since heart rate did not change. By increasing baseline cardiac output for osmoregulatory purposes in seawater via stroke volume, the scope for heart rate can be maintained for further increases in cardiac output through tachycardia during other additional metabolically demanding activities such as swimming or digestion^29^,^39^,^40^,^41^,^42^. While the underlying neurohumoral and/or haemodynamic mechanisms for the apparent increase in cardiac stroke volume in seawater are presently unknown, a study has shown that blood volume and central venous blood pressure decreased in seawater acclimated trout^13^, thus ruling out an increase in cardiac filling pressure as an explanation for the elevated stroke volume. Instead, the observed responses may be explained by some presently unknown neural or endocrine factors (*e.g.* angiotensin II) either stimulating cardiac contractility or influencing the sensitivity of the heart to filling pressure, resulting in a faster and perhaps more complete ventricular emptying during systole^11^,^43^,^44^.

While rainbow trout possess a wide range of potentially energetically costly adaptations allowing them to switch between contrasting osmotic environments, SMR did not significantly change in rainbow trout transitioning to seawater. These results contrast with previous studies on juvenile trout showing an increased metabolic rate in seawater^34^,^45^. However, metabolic responses to changes in salinity depend on life-history stage and body size, *e.g.* due to development of hypo-osmoregulatory mechanisms and a more favorable surface-area-to-volume ratio in larger fish^4^,^46^. It is therefore likely that the lack of metabolic responses in our study can be explained by ontogenetic differences in salinity tolerance and/or the relatively larger size of our experimental animals. Similarly, SMR was unaffected by salinity in similar-sized adult European sea bass (*Dicentrarchus labrax*), suggesting that changing salinity and maintenance of osmotic homeostasis may not cause significant stress or additional metabolic costs at the whole animal level in adult euryhaline teleosts^47^. In fact, although the gill Na^+^/K^+^-ATPase activity increases significantly in response to increasing salinity in trout, the oxygen consumption of isolated gills suggest that energy demands of ion transporting mechanisms constitute a relatively small component (<4%) of the overall metabolism^46^,^48^. However, this does not exclude the possibility that considerable regional increases in metabolism may occur in tissues such as the gastrointestinal tract of fish transitioning to seawater, as oxygen consumption measurements at the whole animal level may not detect such changes. This idea is further supported by recent studies on European flounder (*Platichthys flesus*) displaying a mechanism whereby oxygen delivery to gastrointestinal tissues increases through regional modulation of haemoglobin oxygen affinity in response to increasing salinity^49^. Future studies investigating local gastrointestinal oxygen consumption in fish *in vivo* would be worthwhile as virtually nothing is presently known about the specific gastrointestinal metabolic costs of osmoregulation in fish^17^.

For an anadromous life history to persist it is generally assumed that the benefits of anadromy must outweigh the costs and risks associated with the habitat switch^3^. The present study provides evidence for the importance of gastrointestinal hyperemia during acclimation to a hyperosmotic environment, which may facilitate the transport of absorbed ions and water, as well as supplying oxygen and nutrients to the active gastrointestinal tract. Furthermore, by increasing GBF through increased gastrointestinal pulse volume in seawater, heart rate scope is maintained for additional metabolically demanding activities. In addition, we show that rainbow trout are able to minimize additional costs (or re-prioritize energetic needs) to the extent that we observed no significant change in metabolism during the transition to seawater. This may have positive implications for the relative fitness of a migrating individual, as more aerobic energy may be available for locomotion (*i.e.* foraging and predator avoidance), as well as gonadal and somatic growth^2^.

## Methods

### Experimental animals and holding conditions

Rainbow trout (*Oncorhynchus mykiss* L.), 664 ± 34 g, were obtained from a local hatchery (Antens Laxodling AB, Alingsås, Sweden). Fish were held in 1000 l concrete tanks containing recirculating, aerated freshwater at 10-11 **°**C on a 12:12 h light:dark photoperiod for a minimum period of 3 weeks prior to experimentation. They were fed once a week with dry commercial trout pellets. Animal care and all physiological experimental procedures were performed in accordance with guidelines and regulations approved by the ethical committee of Gothenburg, Sweden (ethical permit 159-2011).

### Surgical procedures

Fish were fasted for approximately 1 week prior to surgery. Individual fish were anaesthetised in freshwater (10 **°**C) containing 100 mg l^−1^ MS222 (ethyl-3-aminobenzoate methanesulphonic acid, Sigma-Aldrich Inc., St. Louis, Missouri, USA) buffered with 200 mg l^−1^ NaHCO_3_. Length and mass were determined and then the fish was transferred to an operating table covered with soft, water-soaked foam. To maintain anaesthesia, gills were continuously flushed with aerated water containing 75 mg l^−1^ MS222 buffered with 150 mg l^−1^ NaHCO_3_ at 10 °C.

**Figure 4:**
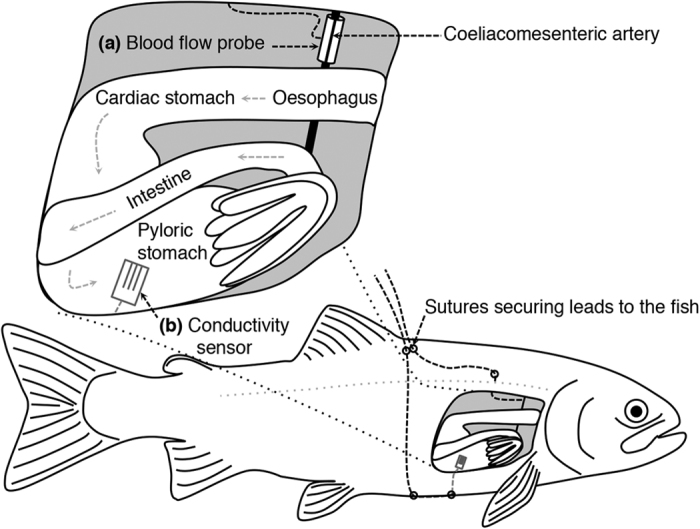
Schematic drawing of surgical instrumentation. **** Rainbow trout (*Oncorhynchus mykiss*) instrumented with a Doppler blood flow probe around the coeliacomesenteric artery for measurements of gastrointestinal blood flow, heart rate and pulse volume (**a**) or a conductivity sensor in the stomach to examine drinking dynamics (**b**). Illustration designed and drawn by Albin Gräns and Jeroen Brijs, respectively.

GBF was measured from the coeliacomesenteric artery (Fig. 4a), which is the first caudal branch of the dorsal aorta and divides progressively to supply the stomach, intestine, liver and gonads^17^. To access this artery, the fish was placed on its left side, and a ~25 mm incision was made ventrodorsally from the base of the pectoral fin^50^. The vessel was dissected free using blunt dissection, ensuring that surrounding blood vessels and nerves remained intact. To measure blood flow, a 20 MHz Doppler flow crystal (Iowa Doppler products, Iowa City, IA, USA) mounted in 1.3–2.0 mm cuffs (depending on the diameter of the artery) was placed around the vessel, approximately 10 mm proximal to the bifurcation from the dorsal aorta^51^. Once the cuff was positioned, the lead was tunneled through the body wall using a sterilized 14G needle, and was subsequently secured to the back of the fish with 3-0 silk sutures. The incision used to access the coeliacomesenteric artery was closed with interrupted stitches using 3.0 prolene sutures (Ethicon Inc., Somerville, New Jersey, USA).

Drinking dynamics of rainbow trout were investigated in a separate group of rainbow trout by surgically implanting a conductivity sensor with an integrated temperature sensor (IST AG, Wattwil, Switzerland) into the pyloric region of the stomach (Fig. 4b). To access the stomach, the fish was placed on the surgical table on its back with the ventral surface exposed. A ~30 mm incision was made in the ventral surface approximately halfway between the pectoral and pelvic fins. A ~10 mm incision was made in the stomach wall and sufficient care was taken to ensure that no gastric fluids leaked out into the peritoneal cavity. The conductivity sensor was gently inserted into the stomach and secured in place by closing the incision with a purse string suture with a 5.0 silk suture (Ethicon Inc., Norderstedt, Germany). The lead from the conductivity sensor exited the peritoneal cavity through the ventral incision, which was subsequently closed with interrupted 3.0 prolene sutures and secured to the back of the fish with 3-0 silk sutures. Following surgeries, fish were transferred to the respirometers and allowed to recover as described below.

### Experimental setup and protocols

The experimental setup consisted of two identical custom-made Perspex respirometers (7.8 L) submerged in a larger experimental tank with recirculating aerated water. Water temperature in the experimental setup was set to 13.5 ± 0.1 **°**C, which was slightly higher than the holding tanks, this prevented any possible temperature deviations between the freshwater or seawater systems during the transition. A digital thermostat connected to a heating unit in a separate header tank controlled the water temperature. To determine any potential effects of surgical instrumentation on SMR during acclimation to seawater, separate groups of uninstrumented and instrumented fish were treated identically and subjected to the following experimental protocols.

Once transferred to the respirometers, all fish were allowed to recover for 72 h prior to continuous recordings of SMR, water temperature and salinity in freshwater. Additionally, cardiovascular variables or stomach conductivity were measured on instrumented fish. After 24 h of recording, the freshwater in the header tank was gradually replaced with seawater (salinity: 30–33 ppt) during a transitional period of approximately 3 h. Fish were then monitored continuously for another 96 h when acclimating to the seawater conditions.

To ensure that the observed responses were due to the actual transition to seawater and not just habituation to the respirometers, extensive control experiments were also performed on both uninstrumented and instrumented fish. These fish went through an identical protocol, but instead remained in freshwater for the entire experimental period. At the end of the experiments, fish were removed from the respirometers and euthanised with a sharp cranial blow.

### Data acquisition

SMR was determined using best practices for intermittent-flow respirometry^52^. Briefly, the oxygen concentration of the water in the respirometer was measured continuously at 1 Hz using a FireSting O_2_ system (PyroScience, Aachen, Germany). Automated flush pumps refreshed the water in the respirometers for 10 min every 15 min period. SMR was calculated from the decline in oxygen concentration in the respirometers between flush cycles. GBF and heart rate were measured with a directional-pulsed Doppler flowmeter (model 545C-4, Iowa Doppler products, USA). All signals were relayed to a PowerLab 8/30 system (ADInstruments, Castle Hill, Australia) and data were collected on a PC using ADInstruments acquisition software Chart^TM^ 5 Pro v7.2.5, at a sampling rate of 10 Hz. For each individual, mean values of GBF were taken from the first 12 h of continuous measurements (*i.e.* after 72 h of recovery) and used to calculate relative GBF for that individual throughout the rest of the experiment. Heart rate was determined by counting the number of blood flow peaks (*i.e.* cardiac systole) per minute. Volume of blood entering the gastrointestinal tract with each heartbeat (pulse volume) was calculated by dividing GBF with heart rate. Stomach conductivity and water salinity were measured with a Thornton M300 transmitter and Thornton 770Max multi-parameter analyzer, respectively (Mettler-Toledo Thornton Inc., Bedford, USA), at a sampling rate of 10 Hz.

### Statistical analysis

Statistical analyses were performed with SPSS Statistics 21 (IBM Corp., Armonk, NY, USA). We used a linear mixed model with the individual fish as the subject variable, measured cardiorespiratory data as dependent variables, treatment (the two experimental groups, *i.e.* fish remaining in freshwater or transitioning to seawater, n = 8 for both groups) as factors, and time as a covariate. In the model we then included the treatments, time and the interaction between these two as fixed effects. Time and intercepts were specified as random effects in a first-order autoregressive (AR1) repeated covariance matrix as we are assuming that each time point is related to its previous values. The subject grouping in this matrix is again specified as individual fish. In order to meet the assumptions of normality and homogeneity of variances, cardiovascular variables (GBF, heart rate and pulse volume) were reciprocally transformed. F- and P- values obtained from the model are reported throughout the text, with significance defined at *P* < 0.05. Unless otherwise specified, all data were presented as means ± s.e.m.

## Additional Information

**How to cite this article**: Brijs, J. *et al*. Increased gastrointestinal blood flow: An essential circulatory modification for euryhaline rainbow trout (*Oncorhynchus mykiss*) migrating to sea. *Sci. Rep.* **5**, 10430; doi: 10.1038/srep10430 (2015).
